# An unusual presentation of anaphylaxis with severe hypertension: a case report

**DOI:** 10.1186/s13256-022-03528-y

**Published:** 2022-08-26

**Authors:** Dumitha Govindapala, Uththara Sachinthanie Senarath, Dasun Wijewardena, Dilini Nakkawita, Chandimani Undugodage

**Affiliations:** 1 Department of Clinical Sciences, Faculty of Medicine, General Sir John Kotelawala Defence University, Ratmalana, Sri Lanka; 2 Department of Paraclinical Sciences, Faculty of Medicine, General Sir John Kotelawala Defence University, Ratmalana, Sri Lanka; 3 Department of Physiology, Faculty of Medical Sciences, University of Sri Jayewardenepura, Gangodawila, Nugegoda, Sri Lanka

**Keywords:** Anaphylaxis, Hypertensive anaphylaxis, Hypertension, Orthostatic intolerance, Case report

## Abstract

**Background:**

Low blood pressure and associated postural symptoms are well-recognized manifestations of anaphylaxis. Nonetheless, anaphylaxis can present with high blood pressure and is rarely reported in the literature. We report an unusual presentation of anaphylaxis with severe supine hypertension and orthostatic intolerance.

**Case presentation:**

A 43-year-old Asian female presented to the emergency department with generalized itching, hives, and postural dizziness after taking a slow-release diclofenac sodium 100 mg tablet. On admission, the patient was tachycardic with a supine blood pressure of 200/100 mmHg. She had urticaria and bilateral rhonchi. A clinical diagnosis of anaphylaxis was made. She was treated with intravenous hydrocortisone and chlorpheniramine, but intramuscular adrenaline was withheld owing to her high blood pressure. She was kept in the supine position, and her vital parameters were closely monitored. Although the respiratory and cutaneous symptoms improved with treatment, her blood pressure remained elevated. Forty minutes later, the postural dizziness recurred as she sat up on the bed and her blood pressure plummeted from 198/100 mmHg to 80/60 mmHg. She was put back in the supine position immediately, and the blood pressure was restored with three doses of intramuscular adrenaline and a fluid bolus. Her postural symptoms completely resolved after adrenaline, but her blood pressure remained elevated. Two weeks after the initial presentation, a diagnosis of essential hypertension was made, which probably had been undetected. In anaphylaxis, where the cardiovascular system is involved, a blood pressure reduction from baseline is expected in patients with preexisting hypertension. Despite cardiovascular involvement, our patients’ blood pressure on presentation to the emergency department was much higher than her pretreatment ambulatory blood pressure, thus making this presentation unusual.

**Conclusions:**

Diagnosis and treatment of anaphylaxis can be delayed in patients presenting with high blood pressure. Postural symptoms should alert the clinician to cardiovascular involvement despite elevated supine blood pressure. Early treatment with adrenaline should be considered in these patients with extreme caution.

## Background

Anaphylaxis is a clinical diagnosis based on physical findings on presentation with or without a history of exposure to an allergen [1]. The clinical manifestations, severity, and sequence of symptoms can vary among individuals depending on the organ involvement [2]. Despite the availability of widely accepted criteria to aid the diagnosis, the early recognition of anaphylaxis can be challenging for clinicians.

Anaphylaxis is characterized by sudden-onset skin or mucosal changes, respiratory or gastrointestinal manifestations, and blood pressure reduction occurring in any combination. Cardiovascular symptoms are potentially life-threatening manifestations of anaphylaxis and are observed more commonly in adults than in children [1]. Hypotension is the key clinical indicator of cardiovascular involvement. Orthostatic intolerance is another manifestation of circulatory instability [1, 2]. In those with preexisting hypertension, a more than 30% reduction from baseline systolic blood pressure indicates cardiovascular involvement [3]. The presence of hypertension at the presentation of anaphylaxis makes the diagnosis extremely challenging. As anaphylaxis is treated with intramuscular (IM) adrenaline, the decision to give adrenaline in the presence of hypertension is equally difficult. Anaphylaxis presenting with hypertension is rarely reported in the literature [4].

We report a case of anaphylaxis presenting with severe hypertension and orthostatic intolerance. This report highlights the importance of suspecting anaphylaxis irrespective of the patient’s blood pressure, early treatment with adrenaline, and maintaining the supine posture until a complete recovery is achieved.

## Case presentation

A 43-year-old Asian female presented to the emergency department with generalized itching, hives, and postural dizziness 1 hour after taking slow-release diclofenac sodium (diclofenac sodium SR) 100 mg tablet for headache. She has a history of migraine, for which she had been taking diclofenac sodium SR. Two months prior to this episode, she had developed itching of her palms after taking a diclofenac sodium SR tablet, which had resolved spontaneously. Apart from one incident of food allergy to northern pilchard, she had no food or drug allergy. She had no history of asthma.

At the onset of her symptoms, the patient had taken 4 mg of chlorpheniramine for itching and continued to do household chores. Although the itching subsided after about 15 minutes, she felt lightheaded and weak on standing, which prompted her to lie down. Her postural symptoms resolved within 5 minutes of lying down. Over the next 20 minutes, she developed a dry cough with difficulty breathing. Her chest symptoms were relieved after taking two puffs of salbutamol from a metered-dose inhaler prescribed for her daughter. Subsequently, as the itching recurred with hives, she decided to seek medical care. The patient again felt dizzy and lightheaded on getting up and therefore remained supine while traveling to the hospital.

On admission to the emergency department, she was conscious and rational, with a pulse rate of 110 beats per minute and supine blood pressure of 200/100 mmHg. Her respiratory rate was 16 breaths per minute and oxygen saturation (SpO_2_) was 99% on room air. Examination revealed skin erythema, cutaneous edema, mild urticaria, and scattered rhonchi in both lungs. The rest of the system examination was normal.

She was given intravenous hydrocortisone 200 mg and chlorpheniramine 10 mg. Her vital parameters were monitored continuously. Intramuscular (IM) adrenaline and intravenous fluid were withheld because of high blood pressure. As the patient was tachycardic, nebulized adrenaline or salbutamol was not given. Her cutaneous and respiratory symptoms improved with treatment, but pulse rate and blood pressure remained persistently high.

Forty minutes later, she felt the need to pass urine and was advised to use a bedpan. However, as the patient insisted on using the washroom, she was asked to sit up on the bed without standing immediately. Her dizziness and lightheadedness recurred within minutes of sitting up, and a dramatic drop in her blood pressure from 198/100 mmHg to 80/60 mmHg was observed. She was put back in the supine position, and her legs were elevated. Intramuscular adrenaline 0.5 mg was administered immediately, and a fluid bolus was given. As the blood pressure remained low (80/62 mmHg), IM adrenaline was repeated twice, 5 minutes apart, until the systolic blood pressure was more than 100 mmHg. The patient was then transferred to the intensive care unit (ICU) for further observation. Her postural symptoms resolved completely after the administration of adrenaline. Her blood pressure fluctuated over the ensuing 24 hours with frequent high readings, and she remained tachycardic. Noteworthily, her blood pressure readings after treatment with adrenaline were not as high as the blood pressure at presentation. There were no hypotensive episodes. All her basic laboratory investigations, including full blood count (FBC), C-reactive protein (CRP), and renal function tests were normal. Serum tryptase was not done as the clinical criteria for the diagnosis of anaphylaxis were met. Two days later, when she was discharged from the hospital, her blood pressure was 165/95 mmHg.

On discharge, a diagnosis of anaphylaxis to diclofenac sodium SR was made with a probable diagnosis of essential hypertension. She was prescribed an adrenaline autoinjector for emergency use and advised not to take diclofenac sodium or other nonsteroidal anti-inflammatory drugs (NSAIDs). Upon reviewing 2 weeks later, a 24-hour ambulatory blood pressure monitoring confirmed that she had previously undiagnosed hypertension. Interestingly, the highest recorded pretreatment systolic blood pressure (BP) was 170 mmHg, which was lower than her blood pressure on admission with anaphylaxis. All relevant imaging and hormonal investigations to identify a secondary cause for hypertension were found to be normal. She was commenced on telmisartan 40 mg and amlodipine 2.5 mg in the night and showed a good response to treatment.

**Fig. 1:**
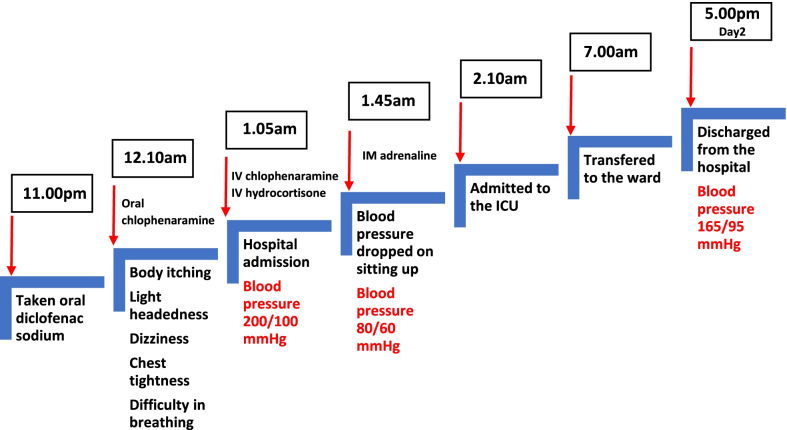
Summary of the sequence of events from the onset of anaphylaxis until the patient was discharged from the hospital

**Fig. 2:**
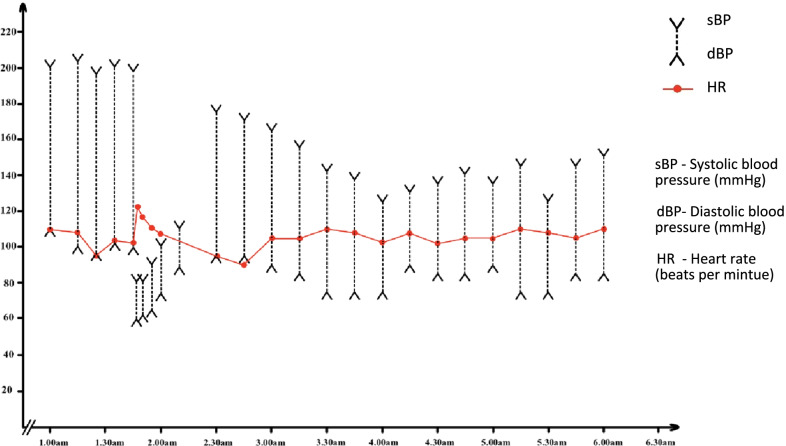
Blood pressure and pulse rate recordings of the patient within the first 6 hours of hospital admission

The timeline of events is summarized in Fig. 1. The cardiovascular observations within the first 6 hours of the hospital admission are depicted in Fig. 2.

## Discussion

Prompt recognition and early treatment with adrenaline are imperative for the appropriate management of anaphylaxis. In the absence of any immediate confirmatory tests, the diagnosis of anaphylaxis is based on pattern recognition. Skin is the commonest organ involved in anaphylaxis (90% of episodes), followed by the respiratory system (70%). Cardiovascular system involvement is recognized among only 10–45% of patients [5].

This patient presented with skin and respiratory symptoms following exposure to a known allergen, making anaphylaxis the most likely diagnosis. She had postural dizziness from the onset of her symptoms suggesting cardiovascular involvement. Once anaphylaxis is triggered by a specific antigen, the mast cells and basophils are activated through different immunological mechanisms. Both mast cells and basophils play a key role in initiating and amplifying the allergic reaction by releasing inflammatory mediators. The pro-inflammatory properties of these mediators lead to increased vascular permeability, vasodilatation, increased glandular secretions, and smooth muscle spasms [6]. The vasodilatation and extravasation of fluid from capillaries cause a mix of distributive and hypovolemic shock in anaphylaxis resulting in hypotension and postural symptoms [3, 7]. Although hypotension is the expected finding with cardiovascular involvement in anaphylaxis, our patient had high blood pressure and postural dizziness. She was previously healthy and had no postural symptoms in the past. Therefore, anaphylaxis with cardiovascular involvement is the most probable explanation for her postural dizziness.

At this patient’s presentation, the recorded high blood pressure could be due to preexisting hypertension, anxiety leading to sympathetic activation, or both. Anaphylaxis-induced high blood pressure is another rare possibility [4]. The sympathetic activation in anxiety may have increased her blood pressure from baseline. However, cardiovascular involvement (as evident by the postural symptoms) should theoretically give rise to a low or reduced blood pressure from baseline. Therefore, her high BP on admission points toward a possibility of anaphylaxis-induced hypertension.

Hypertensive anaphylaxis has been reported previously by Solmazgul *et al*. [4]. Evidence demonstrates that internal compensatory vasopressor mechanisms are activated within minutes of anaphylaxis development, resulting in the release of endogenous vasoactive substances, including epinephrine and norepinephrine, and the formation of angiotensin II [3, 6, 8]. Conversion of angiotensin I to angiotensin II is enhanced by chymase, an inflammatory mediator released during the mast cell degranulation [9]. These endogenous vasoactive substances compensate for vasodilatation and fluid extravasation induced by the inflammatory mediators. Solmazgul *et al*. hypothesized that these initial compensatory mechanisms could be dominant in some patients, resulting in hypertension. This hypothesis was based on a case review of 62 patients who had anaphylaxis, and 8 of them were found to have high blood pressure at presentation [4]. A study on cardiovascular collapse due to anaphylaxis had shown initial vasoconstriction among 10 patients who presented with anaphylaxis [7]. In the same study, early restoration of blood pressure without inotropic agents was observed in 60 patients diagnosed with anaphylaxis [7]. The observations of both studies suggest that in some patients the initial endogenous compensatory vasopressor mechanisms may result in an exaggerated BP response or restore the blood pressure to normal limits. The intrinsic vasopressor mechanisms were likely dominant in our patient, elevating her blood pressure above the baseline.

However, these compensatory mechanisms may not be adequate to maintain the blood pressure when posture changes from supine to upright [10]. The pro-inflammatory mediator-induced vasodilatation and extravasation of fluid from capillaries reduce the venous return to the heart in anaphylaxis. Although the venous return could be compensated on supine posture, the compensatory mechanism may not be sufficient to maintain adequate blood flow to the heart on standing or sitting up, causing orthostatic intolerance [10]. This could explain the postural dizziness in our patient despite the high supine blood pressure.

**Fig. 3:**
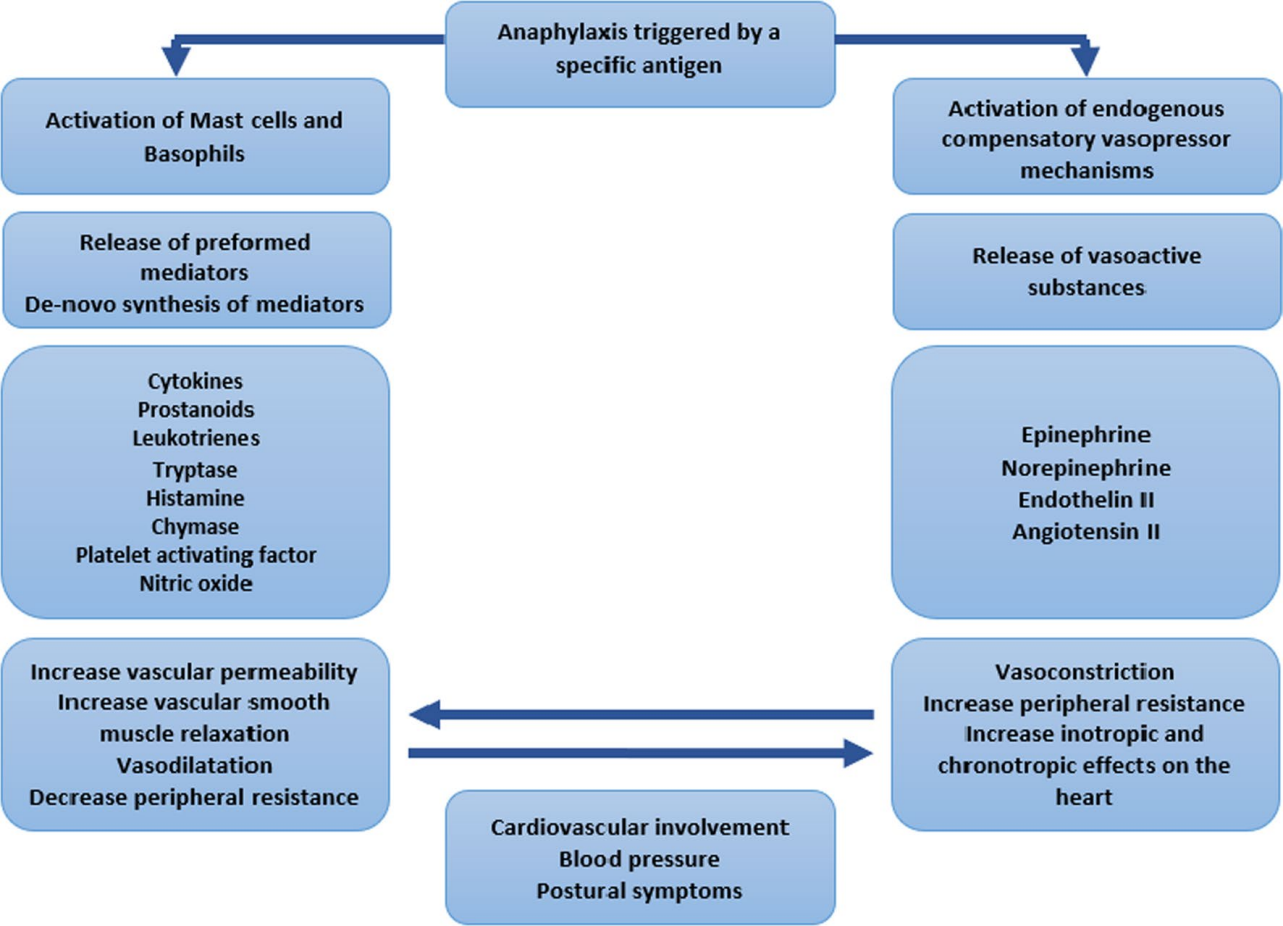
The mechanisms that are activated with triggering of anaphylaxis by an antigen and their effects on the cardiovascular system and blood pressure of  a patient

Figure 3 illustrates the activation of compensatory vasopressor mechanisms and the determinants of blood pressure in anaphylaxis.

The safety of adrenaline administration in the presence of high blood pressure is an important concern. Adrenaline is the drug of choice in the management of anaphylaxis as laryngeal edema and vasodilatation are responsive only to adrenaline [3]. Both are life-threatening conditions, and a delay in administering adrenaline makes subsequent management difficult. On the other hand, adrenaline can give rise to extremely high blood pressure leading to intracranial hemorrhages and fatal arrhythmias [11]. In the study by Solmazgul *et al*., two of the eight patients who had hypertensive anaphylaxis were treated with IM adrenaline. Their pretreatment systolic BP was 150 mmHg, and both the anaphylaxis and hypertension recovered completely without adverse reactions after the administration of IM adrenaline. Others recovered without IM adrenaline [4]. As per another medical communication, a patient who developed anaphylaxis with high BP and tachycardia following an injection of wasp venom also recovered with oral steroids and antihistamines without adrenaline [12].

We did not administer adrenaline at the outset in our patient, but it was given immediately upon the drop in blood pressure. The initial delay in administering adrenaline is likely the reason for her near-fatal collapse after 2 hours from the onset of her symptoms. Delayed use of adrenaline is associated with increased severity and fatalities in anaphylaxis [13]. Furthermore, patients with anaphylaxis who are normotensive at presentation can later develop hypotension [11]. IM adrenaline has been shown to reduce the risk of subsequent hypotension without any adverse events in such patients [11].

Glucagon is recommended as a second-line agent for patients with anaphylaxis who are unresponsive to adrenaline [1].

Glucagon can be considered as an alternative therapy for adrenaline in the presence of high BP. However, positive inotropic effects of glucagon can precipitate severe hypertension. Though this is a less common side effect of the drug, more scientific data are required on the use of glucagon in hypertensive anaphylaxis. The benefit of glucocorticosteroids in the acute management of anaphylaxis is controversial [14, 15]. Although first-generation antihistamines are used to relieve cutaneous symptoms, their role is limited in the management of anaphylaxis (16). Therefore, we suggest using adrenaline despite high blood pressure in patients with anaphylaxis, especially in the presence of postural symptoms; however, this should be done with extreme caution.

## Conclusion

Anaphylaxis can present with normal, low, or elevated blood pressure. The scarcity of corroborating reports in the literature on anaphylaxis presenting with high blood pressure makes this entity unfamiliar to clinicians, leading to underdiagnosis and delayed treatment. Postural symptoms despite high blood pressure should alert the clinician to cardiovascular involvement, and close monitoring in the supine position is essential. Early treatment with adrenaline should be considered in these patients with extreme caution. Further studies are required to better understand the pathophysiology and management of anaphylaxis associated with high blood pressure and orthostatic intolerance.

## Data Availability

The supporting data and material of this case report are not publicly available owing to risk of breach of patient confidentiality but are available from the corresponding author on reasonable request. The patient’s de-identification will be maintained in sharing the data.

## Abbreviations

BP: Blood pressure
CRP: C-reactive protein
ED: Emergency department
FBC: Full blood count
ICU: Intensive care unit
IM: Intramuscular
NSAIDs: Nonsteroidal anti-inflammatory drugs
SR: Slow release
